# Efficient preparation of nanocatalysts. Case study: green synthesis of supported Pt nanoparticles by using microemulsions and mangosteen peel extract†

**DOI:** 10.1039/d2ra04134k

**Published:** 2022-11-30

**Authors:** Riny Yolandha Parapat, Michael Schwarze, Alwin Ibrahim, Minoo Tasbihi, Reinhard Schomäcker

**Affiliations:** Chemical Engineering Department, Institut Teknologi Nasional Bandung PHH. Mustopha 23 40124 Bandung Indonesia rinyyolandha@itenas.ac.id; Department of Chemistry, Technische Universität Berlin Straße des 17. Juni 124 10623 Berlin Germany

## Abstract

Greener nanocatalyst synthesis is growing in importance, especially when using scarce noble metals such as platinum (Pt) as the active metal. In the synthesis process presented herein, we utilized extract of mangosteen peel as a green reductant and found that it produces Pt nanoparticles (NPs) with high activity. The supported Pt NPs were synthesized *via* thermos-destabilization of a mangosteen extract microemulsion and subsequently tested with α-methyl styrene (AMS) hydrogenation at SATP. Additionally, we optimized the green synthesis of the supported Pt nanocatalyst (NPs) in terms of their synthesis yield and catalytic activity using the approaches of full factorial design (FFD), central composite design (CCD), and response surface methodology (RSM). In comparing the results of single and multiple optimization, it was found that for the single optimization, the synthesis yield of supported Pt NPs could be increased from their average value of 78.9% to 99.75%, and their activity from 2136 to 15 600 μmol s^−1^ g_Pt_^−1^. The results of multiple response optimization to the yield and activity are 81.71% and 8255 μmol s^−1^ g_Pt_^−1^, respectively. The optimization approach presented in this study is suitable for similar catalyst synthesis procedures where multivariate responses are sensitive to a number of experimental factors.

## Abstract 2

The optimal approach to efficient nanocatalysts synthesis is to optimize both the yield and activity simultaneously. The optimization principle in this paper can also be more broadly applied to other multivariable processes.

## Graphical abstract

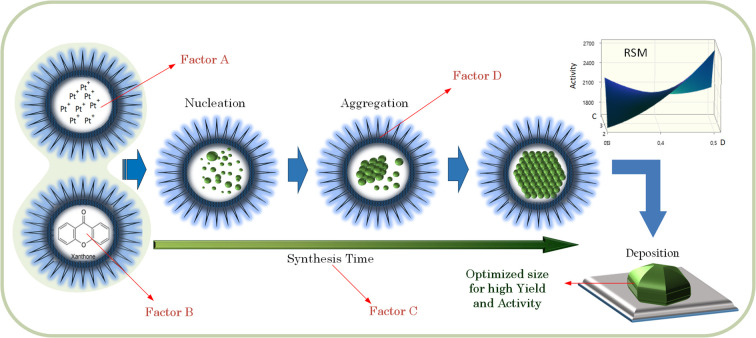

## 1. Introduction

Homogeneous and heterogeneous catalysis plays vital roles in the chemical industry to ensure good product selectivity; both systems have their advantages and disadvantages. Homogeneous catalysts have high activity and selectivity, but purification is needed to recover them from the product stream. Heterogeneous catalysts, on the other hand, are more stable and are easier to recover, but have relatively lower catalytic activity and require longer reaction times.^1,2^ Supported nanocatalysts present themselves as an attractive compromise, as they have demonstrated both the activity and selectivity of homogeneous catalysts while also possessing the facile separability and longevity of heterogeneous catalysts.^3,4^

Designing nanoparticles (NPs) as nanocatalysts is a challenging endeavor due to the tunability of their size and morphology, which creates a seemingly limitless space of multivarious features.^5,6^ Synthesis of NPs by microemulsion method remains a reliable and well-studied approach because the size and shape of the NPs can be well designed and preserved inside the micelles present in the microemulsion.^7–10^ In synthesizing of NPs, microemulsions are often prepared using reductants such as borohydride (NaBH_4_) and hydrazine (N_2_H_4_), which are hazardous to both human and environmental health.^11–13^

Therefore, an approach that makes use of environmentally benign, or “green”, reductants in the microemulsion for NPs synthesis would render the process more sustainable. Finally, the nanoparticle catalysts need to be stabilized *via* attachment support materials. In our previous work, we have demonstrated a novel technique of depositing NPs on support materials *via* a process called thermo-destabilization of microemulsions.^14^

Optimizing catalytic activity is a primary objective when developing NP synthesis procedures. Our approach uses four synthesis variables, or factors, to optimize the production of supported NPs. These factors were selected based on our previous study, which illustrated how the activity of the produced NPs was influenced by the (i) metal content of the precursor, (ii) amount of reductant, (iii) reaction time, and (iv) surfactant concentration.^10,12,14^ By conducting the experiments according to a full factorial design (FFD), and then using a central composite design (CCD) as well as a response surface methodology (RSM) to optimize the desired response (activity), we obtained the theoretical values for each of the four factors that corresponds to maximum activity. However, an additional concern in the production of nanocatalysts at large scale is the maximizing of synthesis yield, especially when dealing with expensive precursors (*e.g.*, noble metals). Besides being economically unfavorable, a low catalyst yield is also indicative of a wasteful and potentially environmentally harmful practice. Therefore, the optimal approach to efficient NP synthesis is to optimize both the yield and activity of the nanocatalysts.

Prior attempts to optimize NP synthesis have been reported utilizing different approaches with varying optimization goals, but most primarily focus on a single response: either optimizing the yield or the activity.^15–18^ To the best of the authors' knowledge at the time of writing this article, simultaneous optimization of both synthesis yield and activity of produced NPs is currently not present in the literature. For example, a recent, comprehensive review by Rodrigues *et al.* concerning the optimization of noble metal nanoparticles as nanocatalysts did not reveal any prior dual-optimization approaches.^17^

The study herein presents the optimization of the green synthesis of supported Pt NPs. In the experimental design, using only a 2^*k*^ factorial approach typically produces a first-order model, which shows a lack of fit. The CCD method,^19,20^ the most widely used experimental design for second-order models, is therefore needed to generate quadratic models suitable for RSM. RSM was employed to optimize the responses (synthesis yield and activity of NPs), which are influenced by several independent variables (factors). Screening trials published in our previous study uncovered several variables that affect the responses, which include the following: metal amount (*A*), reductant amount (*B*), reaction time (*C*), and the mass fraction of surfactant in the microemulsion (*D*). By applying experimental designs including FFD, CCD, and RSM, we were able to predict what factor values maximize our system's responses. The optimization principle in this paper can also be more broadly applied to other multivariable processes including, but not limited to, catalyst synthesis.

## 2. Methods

### 2.1 Chemicals

A microemulsion synthesis approach was utilized. The water phase containing the metal salt was prepared by dissolving hexachloroplatinic acid hydrate (99.9%, Sigma-Aldrich) in deionized water. 250 mg of natural reducing agent consisting of either mangosteen peel (MS, Mastin Borobudur, Indonesia), clove (CL, Supa Sidoarjo, Indonesia), grape seed (GS, Glory Feel, Hamburg, Germany), dried green tea leaf (Pucuk Bola, Indonesia), or arabica coffee (Tchibo, Germany) were dissolved in 4 g of deionized water and extracted for 1 hour at 70 °C, and then centrifuged to remove the solids. For the surfactant, co-surfactant, and oil phase, Triton X-100 (100%, Sigma-Aldrich), 1-pentanol (≥98%, Carl-Roth), and cyclohexane (≥99.5%, Carl-Roth) were used, respectively. Neutral-Al_2_O_3_ (155 m^2^ g^−1^, Brockman 1, Sigma-Aldrich) was used as the support material for the nanocatalysts. Acetone (99,9%, Carl-Roth) was used to wash the supported nanocatalysts. α-Methyl styrene (99%, Sigma-Aldrich) was used as the substrate, and methanol (≥99.9%, Carl-Roth) was used as the solvent for the catalytic AMS hydrogenation test.

### 2.2 Synthesis of supported Pt-nanocatalysts

**Fig. 1:**
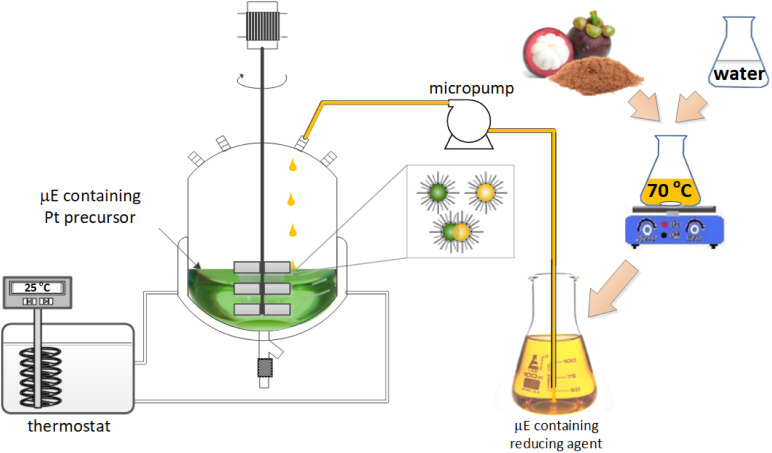
Schematic illustration of the green synthesis of supported Pt NPs with microemulsions.

The reactor setup used for nanocatalyst synthesis is schematically presented in Fig. 1. The synthesis reaction took place in a double-walled glass reactor with a volume of 200 mL. Microemulsions containing metal precursors were first introduced into the reactor, followed by a slow injection of the microemulsion containing the natural reductants at a flow rate of 0.2 μL s^−1^ controlled *via* a micropump. The resulting mixture was stirred at 700 rpm at room temperature for 1 hour. After stirring, a deposition process was carried out by adding 5 g of support material (Al_2_O_3_) into the reactor. Stirring recommenced at 700 rpm, and the reactor was recirculated with water from a thermostat at a temperature of 55 °C. After 2 h of the deposition process, stirring and heating ceased, and the produced nanocatalyst was collected from the reactor and washed three times with pure acetone. The clean nanocatalyst was calcined at 300 °C for 2 h. ICP-MS was used to measure the amount of deposited Pt NPs on the support material. The following equation (eqn (1)) calculates the synthesis yield of supported Pt NPs:1
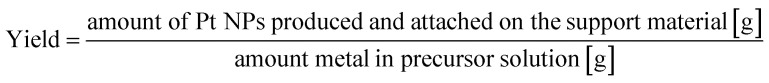


### 2.3 Catalytic testing of supported Pt-nanocatalysts

**Fig. 2:**
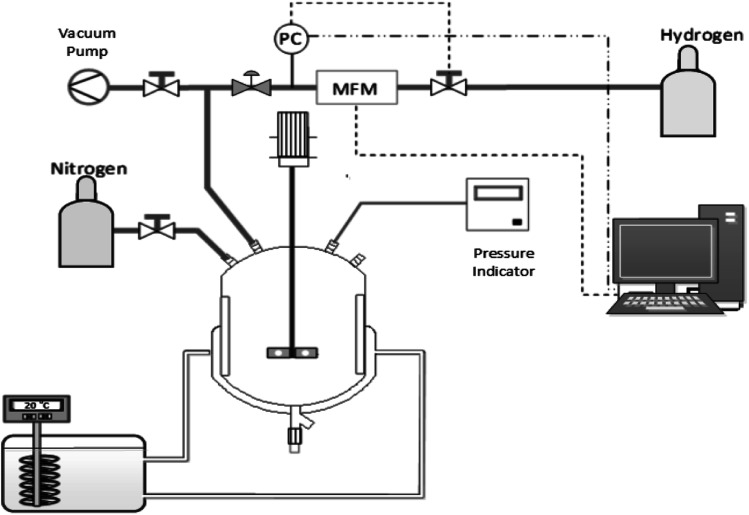
Schematic of the AMS hydrogenation setup.

To determine their activities, the supported Pt NPs were tested *via* α-methyl styrene (AMS) hydrogenation. The reaction was carried out in a double-walled glass reactor with a volume of 200 mL at 25 °C, an initial hydrogen pressure of 1.1 bar, with the solution stirred at 1200 rpm (Fig. 2). The hydrogenation process was considered complete when no more hydrogen consumption was observed, which was monitored *via* an Excel program. The following equation (eqn (2)) calculates the activity of the Pt nanocatalyst, where the initial rate is defined as the rate calculated during the first 5 minutes of reaction:2
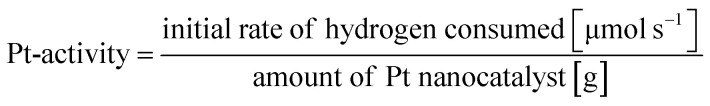


### 2.4 Nanocatalyst synthesis optimization

In our optimization procedure, a sequence of experiments was first conducted to obtain the empirical models representing the fitted response equations, which are subsequently used to estimate the correct system response. The CCD from 2^*k*^ factorial design with four factors (*k* = 4) and RSM were used to optimize the catalyst synthesis procedure and evaluate the effect of different variables and their interactions on the synthesis process. The factors determined previously that potentially affect the response along with their low and high levels are presented in Table 1. In our experiments, we fixed the value of *α* to 0.92 and varied *γ*, where *α* = *m*_oil_/(*m*_oil_ + *m*_water_) and *γ* = *m*_surfactant_/(*m*_oil_ + *m*_water_ + *m*_surfactant_). The optimization with CCD and RSM were performed with Minitab® software.

**Table 1:** Factors and levels in the synthesis of supported NPs for 2^4^ factorial design

Level	Factor			
	Mass of Pt in precursor (mg), *A*	Mass of green reductant (mg), *B*	Reaction time in synthesis process (h), *C*	Mass fraction of surfactant (*γ*), *D*
Low (−)	5	125	1	0.3
High (+)	25	250	2	0.5

## 3. Result and discussion

**Fig. 3:**
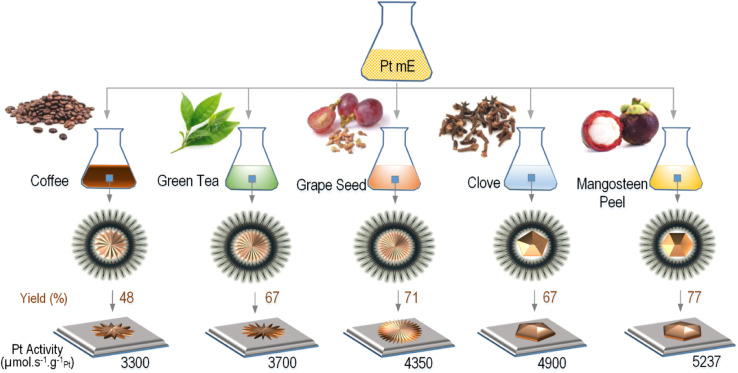
The catalytic activity and production yield of supported Pt NPs produced *via* thermos-destabilization of microemulsions and using different kinds of green reductants. The synthesis was done at the same reaction condition and reactants composition in the microemulsion. The shapes of NPs are illustrated based on the results of our SEM and TEM investigations.

Before performing process optimization, we first screened five different green reductants including coffee, green tea, clove, grape seed, and mangosteen peel extracts used to produce Pt-NPs in order to see which generated the highest synthesis yield and catalyst activity. Fig. 3 shows the comparison of the yield and activity of Pt NPs corresponding to each natural reductant. It can be seen that Pt NPs reduced by the mangosteen peel extract had superior synthesis yield and catalytic activity compared to the other natural reductants, which is in agreement with the results reported by Schwarze *et al.* when producing Pt NPs on TiO_2_ as catalyst for water splitting.^7^ For this reason, we used the mangosteen peel extract to produce Pt NPs.

The mangosteen peel extract may serve as a reductant because it contains xanthones, a phenolic compound derived from diphenyl-γ-pyrone, which has a reduction potential of −1.69 V.^21^ As a strong antioxidant, xanthones neutralize Pt cations by giving up some of their own electrons.^22,23^ The extraction of mangosteen peel has been previously carried out by Zarena *et al.*, using a supercritical carbon dioxide process at pressures of 20–30 MPa and temperatures of 40–60 °C.^24^ They reported that the mangosteen peel contains several types of xanthones with different chemical structure, but the major substance was identified as α-mangostin (∼43%), followed by garcinone E and gartanin. It was observed that γ-mangostin showed the lowest enrichment.

**Fig. 4:**
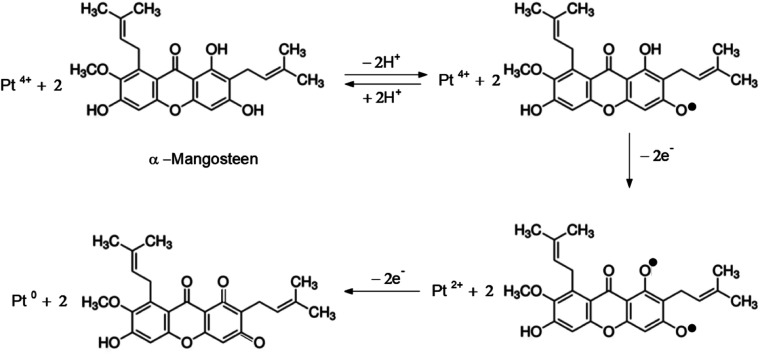
Scheme of Pt^4+^ reduction pathway by α-mangosteen.

In our case, we extracted mangosteen peel by using water as the solvent at 70 °C for 30 min. With this mild extraction (compared to the method and condition explained above), we assume that only little amount of the major substance (α-mangostin) would be extracted. In our experiment, we used only the mass of mangosteen peel as the variable (not the concentration of xanthones), so that the results could be obtained proportionally. In other words, we surmised that the amount of xanthones in the extract of 250 mg mangosteen peel would be double that of 125 mg. Assuming that the main content of our mangosteen peel extract is α-mangosteen, we propose the Pt^4+^ reduction pathway as shown in Fig. 4.

**Fig. 5:**
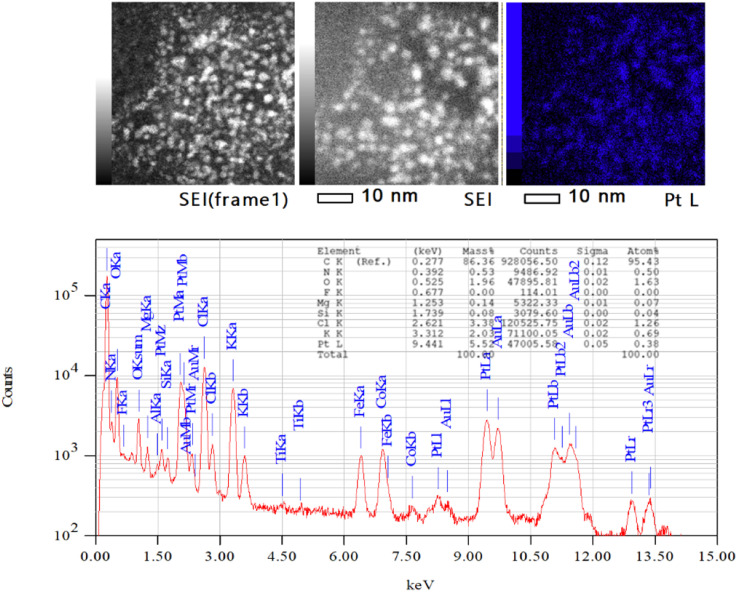
The EDX analysis of the Pt NPs before deposited on the alumina using JEM-ARM300F2(WS)ED 300.0 kV, real time: 1244.22 s, dead time: 3%, and counting rate: 1659 cps.

To verify that the Pt NPs have been formed before deposition on the alumina support, we investigated the sample with TEM and EDX (Fig. 5). The corresponding TEM images are the Pt NPS inside of the microemulsions. This was verified by the presence of Pt peaks in the EDX analysis, which indicates that Pt NPs are formed after the synthesis process. The complete TEM and EDX analysis is given in the ESI.†

**Fig. 6:**
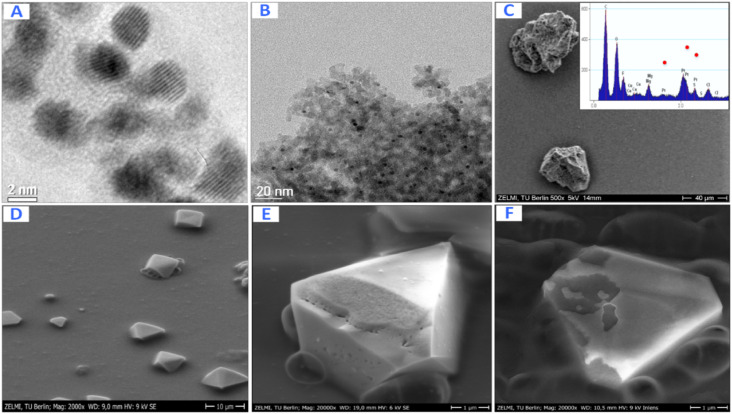
Pt-NPs synthesized using mangosteen peel extract as the reductant: TEM images of Pt NPs inside the reverse micelles (A) and after they are deposited on the support Al_2_O_3_ (B). SEM images of Pt@Al_2_O_3_ (C) and Pt NPs formed without using microemulsions with different magnification (D–F). Inset: EDX of corresponding Pt@Al_2_O_3_.

The shape and the size of Pt-NPs synthesized using mangosteen-peel extract as the reductant are displayed by the TEM and SEM images in Fig. 6. The size of Pt NPs while inside the reverse micelles (Fig. 6A) and after they are deposited on the support Al_2_O_3_ (Fig. 6B) is about 3–4 nm. The SEM images of the synthesized Pt-NPs without using microemulsions (Fig. 6D and E) indicate that the structures of the NPS have many defects that increase the catalyst's activity. The inset shows the EDX of corresponding Pt@Al_2_O_3_ that verifies the presence of Pt NPs.

**Fig. 7:**
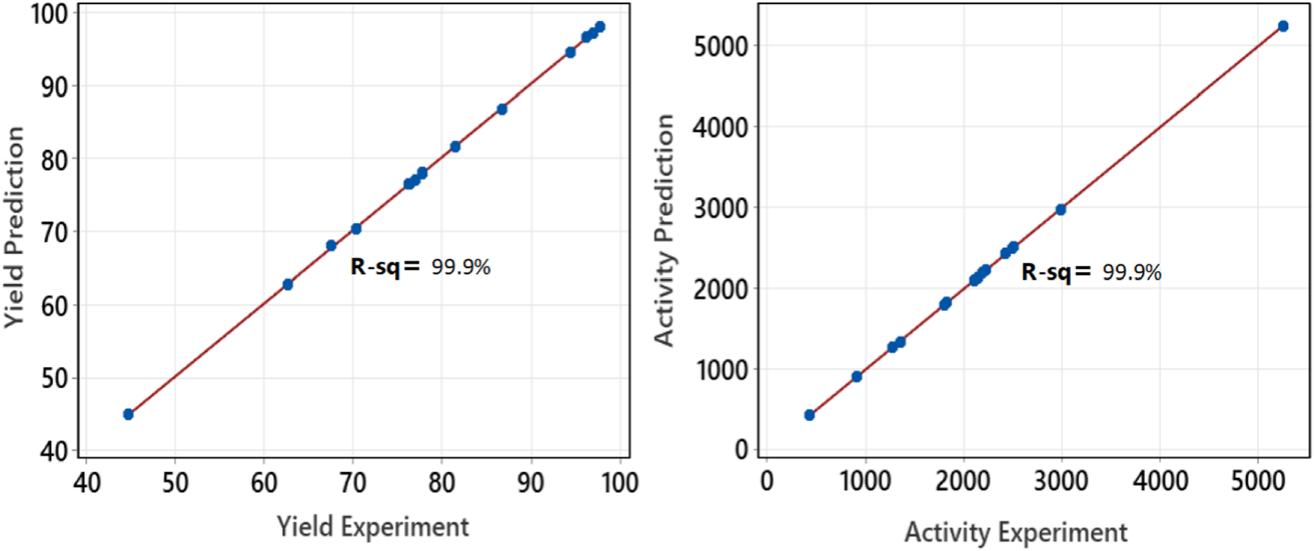
Plots of experiment response *versus* prediction response.

After conducting the series of experiments based on the factorial design shown in Table 1, the data were collected to calculate the yield and Pt-activity using eqn (1) and (2). The activity of the produced supported NPs was tested with a standard reaction: hydrogenation of α-methyl styrene (AMS) at 25 °C and 1.1 bar. To proceed with the optimization, we extended the data according to the CCD matrix with alpha = 2 by using regression equations (eqn (3) and (4)). Table 2 presents the responses which were calculated based on the factorial regression equation confirmed by an *R*-square equal to 99.9% (Fig. 7). This indicates that the regression model is reliable enough to predict the values of responses for the CCD matrix.

3 Yield = −134.9 + 5.052*A* + 1.607*B* + 212.6*C* + 517.6*D* − 0.03820*AB* − 8.202*AC* − 12.77*AD* − 1.406*BC* − 3.687*BD* − 512.1*CD* + 0.05014*ABC* + 0.07334*ABD* + 21.26*ACD* + 3.322*BCD* − 0.1189*ABCD*

4 Activity = 15087 − 699.8*A* − 102.1*B* − 7919*C* − 42842*D* + 5.292*AB* + 557.7*AC* + 2094*AD* + 61.75*BC* + 334.1*BD* + 25615*CD* − 3.832*ABC* − 16.40*ABD* − 1652*ACD* − 191.9*BCD* + 11.26*ABCD*

**Table 2:** Yield and activity of supported Pt NPs

Run	Factor				Response	
	Pt amount (*A*)	Red. amount (*B*)	Time (*C*)	Gamma (*D*)	Yield (%)	Activity (μmol s^−1^ g_Pt_^−1^)
1	15	187.5	2	0.4	84.34	2077
2	25	125	2	0.5	77.88	427
3	15	187.5	1.5	0.4	80.30	2149
4	25	125	2	0.3	96.54	2217
5	15	250	1.5	0.4	78.08	2469
6	15	187.5	1.5	0.4	80.30	2149
7	15	187.5	1.5	0.4	80.30	2149
8	25	250	1	0.3	44.79	1827
9	5	125	1	0.5	86.69	2099
10	5	125	2	0.5	94.48	2495
11	15	125	1.5	0.4	82.52	1829
12	5	250	1	0.3	76.98	2427
13	15	187.5	1.5	0.5	80.42	2136
14	5	250	2	0.3	97.87	2981
15	25	250	1	0.5	76.36	1277
16	15	187.5	1.5	0.4	80.30	2149
17	25	125	1	0.3	76.38	1795
18	15	187.5	1.5	0.4	80.30	2149
19	5	125	2	0.3	76.32	2505
20	25	125	1	0.5	70.36	902
21	25	250	2	0.3	91.04	1353
22	5	250	2	0.5	62.65	2494
23	15	187.5	1.5	0.3	80.17	2162
24	25	250	2	0.5	77.94	2146
25	25	187.5	1.5	0.4	76.41	1493
26	5	250	1	0.5	96.99	5249
27	15	187.5	1	0.4	76.26	2221
28	15	187.5	1.5	0.4	80.30	2149
29	5	187.5	1.5	0.4	84.18	2805
30	5	125	1	0.3	81.48	2190
31	15	187.5	1.5	0.4	80.30	2149

**Fig. 8:**
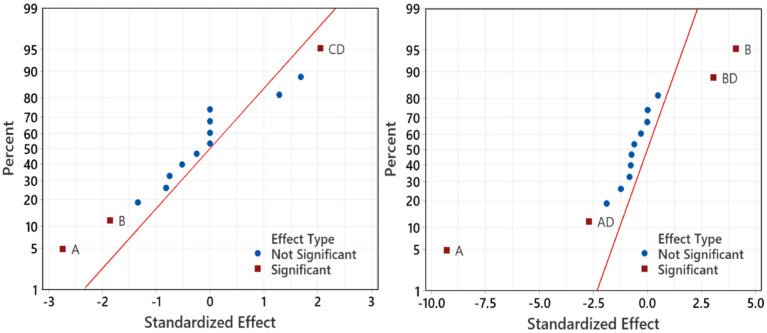
Normal plots of the standardized effects for yield (left) and Pt-activity (right) which show the significant effects of the factor and factor interactions to the response.

Fig. 8 shows the significant factors that influence the responses using the normal plots of the standardized effects. It can be seen clearly that the significant factors affecting the yield and Pt-activity are the same, *i.e.*, the amount of Pt in the precursor (*A*) and the amount of the mangosteen peel (*B*). This is in agreement with the results that have been reported by previous related studies.^10,14,17,25,26^Fig. 8 also shows that the interaction between factors *C* and *D* (*CD*) significantly affects the synthesis yield, while interactions of *AD* and *BD* affect the Pt-activity.

**Fig. 9:**
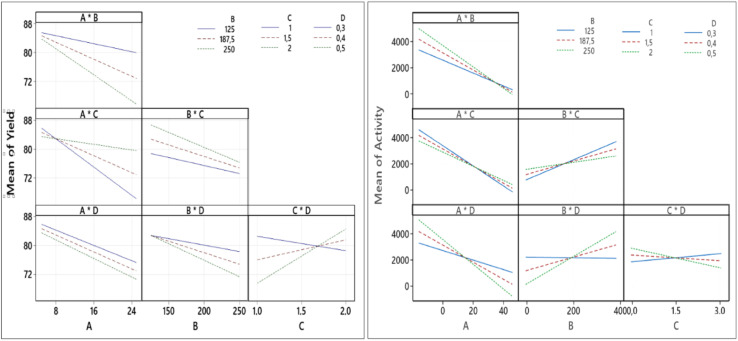
Factor interaction plots for the response of yield (left) and Pt-activity (right).

The quality of factors interaction are confirmed by Fig. 9 which displays the entire interaction plots for the yield and Pt-activity. As we can see clearly in Fig. 9 (left), the slope of intersecting lines of the *CD* interaction is greater than those of other interactions. This indicates that the effect of synthesis time (factor *C*) on synthesis yield is dependant on the size of the micelles (factor *D*) in which the formation of NPs takes place. These results are also confirmed by the Anova tables in the ESI,† which confirm the significant factors.

We note here that the complex chemicals in the extract of mangosteen peel did not interfere or affect the activity of the produced catalyst because we ensured that there were no more organic species, such as xanthones, remaining on the surface of the catalyst by washing the catalyst 3 times with pure acetone, followed by the calcination at 300 °C for 2 h. The absence of any residual organic species was confirmed by the FTIR analysis (Fig. S2 in ESI†). The results show no peak of organic matter on the Pt/Al_2_O_3_, which indicates that the catalyst was already clean. We also note that the support material (Al_2_O_3_) did not play a role in the catalysis because it is inert and very resistant to reduction.^27^ Control experiments were conducted by testing the support in the testing reaction (AMS hydrogenation) and no conversion was detected.

**Fig. 10:**
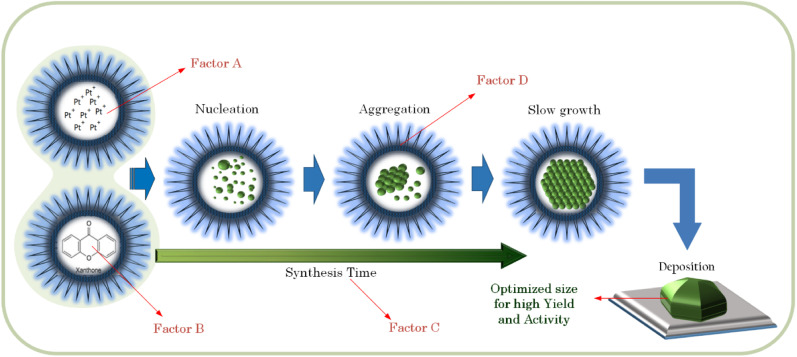
Schematic illustration of a Pt NP formation in the microemulsions and its deposition on the support. Factors *A*, *B*, *C* and *D* are the factors that can influence the yield and activity of the supported Pt NPs.

In the case of the activity, the gradients of the intersecting lines of *AD* and *BD* interactions are more contrast than the other interactions, as shown in Fig. 9 (right). This implies that the effect of either the amount of Pt in the precursor (factor *A*) or the amount of reductant (factor *B*) on the Pt-activity strongly depends also on how big the size of micelles (factor *D*) in which the formation of NPs takes place is. In the microemulsion method, the NPs size can be controlled by the surfactant concentration (factor *D*), the amount of metal precursor (factor *A*), and the amount of reductant (factor *B*).^28–30^ Another influential factor in the activity is the degree of NP dispersion on the support material. If the agglomeration occurs, the nanocatalyst will be less active due to a loss in contact area.^31,32^ Therefore, the factors setting influencing the synthesis of Pt NPs must be determined in order to optimize the size. If the size of the Pt NPs is too big, there will be more ineffective Pt atoms caused by agglomeration, as illustrated in Fig. 10. It has also been reported by Garlyyev *et al.* that the size of Pt NPs needs to be optimized to enhance the activity.^33^ The results of optimization in this study may indicate that the size of the synthesized Pt NPs has been optimized.

**Fig. 11:**
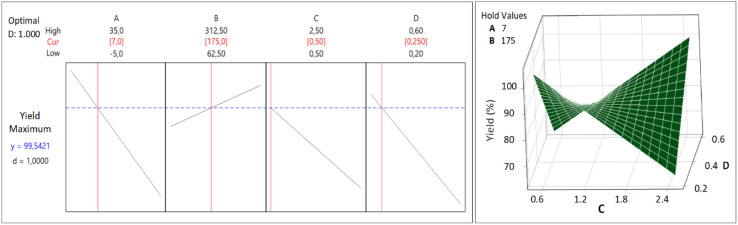
Response optimization (left) and surface plot (right) for the Yield of supported Pt-NPs.

**Fig. 12:**
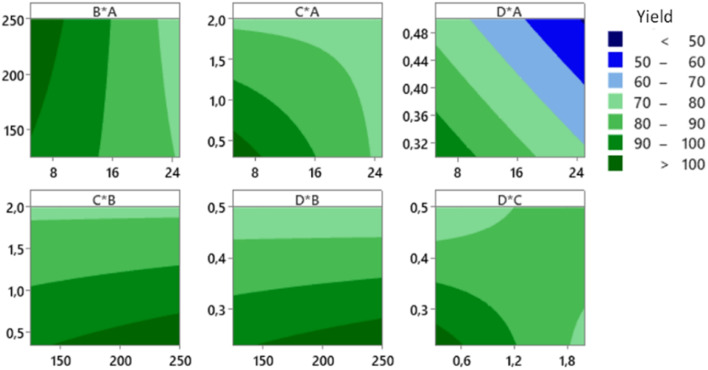
Contour plot of the production yield of supported Pt NPs.

The CCD was applied to get an optimum yield, and the optimization with RSM was executed using Minitab® software to find factors setting that optimize the yield. The optimization result of the synthesis yield of supported Pt NPs using CCD and RSM is shown in Fig. 11 (left), where the predicted optimum yield is 99.54%. The value of desirability (*d*) is 1.0, which indicates that the optimization is effective. This result is confirmed by the surface plot in Fig. 11 (right) and the contour plot in Fig. 12, which shows that the value of *A*, *B*, *C*, and *D* that produce optimum yield are found in the area of high yield (90–100%). The predicted optimum yield was validated by synthesizing supported Pt NPs using the optimized *A*, *B*, *C*, and *D*. The result shows that the validated yield is 99.75% (Table 3).

**Table 3:** Result of the single response optimization for the yield and activity of nanocatalyst

Factor	Lower limit	Upper limit	Optimized yield (%)			Optimized activity (μmol s^−1^ g_Pt_^−1^)		
			Factor	With RSM	Validated	Factor	With RSM	Validated
*A*: Pt in precursor (mg)	5	25	7	99.54	99.75	3.5	16 300	15 600
*B*: green reductant (mg)	125	250	175			375		
*C*: synthesis time (hr)	1	2	0.5			0.5		
*D*: surfactant fraction	0.3	0.5	0.25			0.7		

**Fig. 13:**
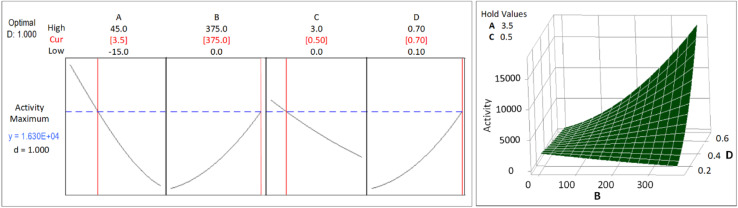
Response optimization (left) and surface plot (right) for the activity of supported Pt NPs.

**Fig. 14:**
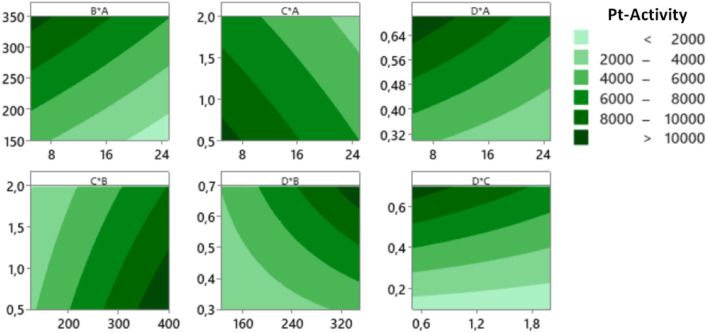
Contour plots of the catalytic activity of supported Pt NPs.

As mentioned previously, the activity of the nanocatalyst is an additional response that needs to be optimized. The optimization results of Pt-activity were obtained using the same procedure applied to optimize the yield. The optimization results of the Pt-activity using CCD and RSM are shown in Fig. 13 (left), where the predicted optimum activity is 16 300 μmol s^−1^ g_Pt_^−1^. The value of desirability (*d*) is 1.0, indicating the optimization is acceptable. The validation of the predicted optimum activity shows that the validated value of the activity is 16 100 μmol s^−1^ g_Pt_^−1^ (Table 3). This result is confirmed by the surface plot in Fig. 13 (right) and the contour plot Fig. 14, which shows that the value of *A*, *B*, *C*, and *D* that produce optimum activity are found in the area of high activity (>10 000). The differences between the predicted and the experiment (validated) values of the optimized yield and Pt-activity are 0.21% and 1.2%, respectively.

As we already see from Table 3, the yield can be optimized to 99.54%, where the corresponding factors of *A*, *B*, *C*, and *D* are 7, 175, 0.5, and 0.25, respectively. However, if these factors are used in the RSM to predict the Pt-activity, we only witness a value of 2100 μmol s^−1^ g_Pt_^−1^. Likewise, for the activity, the optimized value shown in Table 3 can reach 16 300 μmol s^−1^ g_Pt_^−1^ with the factors setting of *A*, *B*, *C*, and *D* are 3.5, 375, 0.5, and 0.7, respectively. The predicted synthesis yield using those factors is also low, only 46.61%. Therefore, for the best result, the yield and activity of the catalyst must be optimized at the same time. However, the way to find the factors setting that can give the optimum values of both yield and activity is rather challenging because there are several possibilities on both surfaces. The automatic finding tool available in Minitab used to execute the RSM for multiple responses optimization generated the results are not presentable because both values are below their average values.

**Fig. 15:**
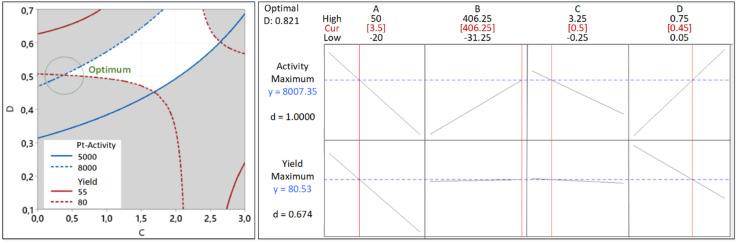
The results of multiple response optimization for the synthesis yield and Pt-activity. The overlaid contour plots of the yield and activity where their intersections indicate the possible location of the optimum values (left). The graphical results of the multiple response optimization using RSM (right).

For this reason, we must find the intersecting lines between the high yield and high activity manually. The first step is to build the contour plots of both responses. To construct the suitable contour plots, we need to use Fig. 9 to see which factors give strong interactions between yield and activity. As apparent in that figure, the CD interaction significantly affects both responses. Fig. 15 (left) shows the point of intersecting lines between the two contours for the yield and activity. Using this maximum point, we optimized both responses using the RSM in Minitab and found the value of *A* and *B* that can give the maximum yield and activity. The graphical RSM optimization of both yield and activity is shown in Fig. 15 (right). The displayed results are better than those of single response optimizations because the values of both yield and activity are higher than their average values.

**Fig. 16:**
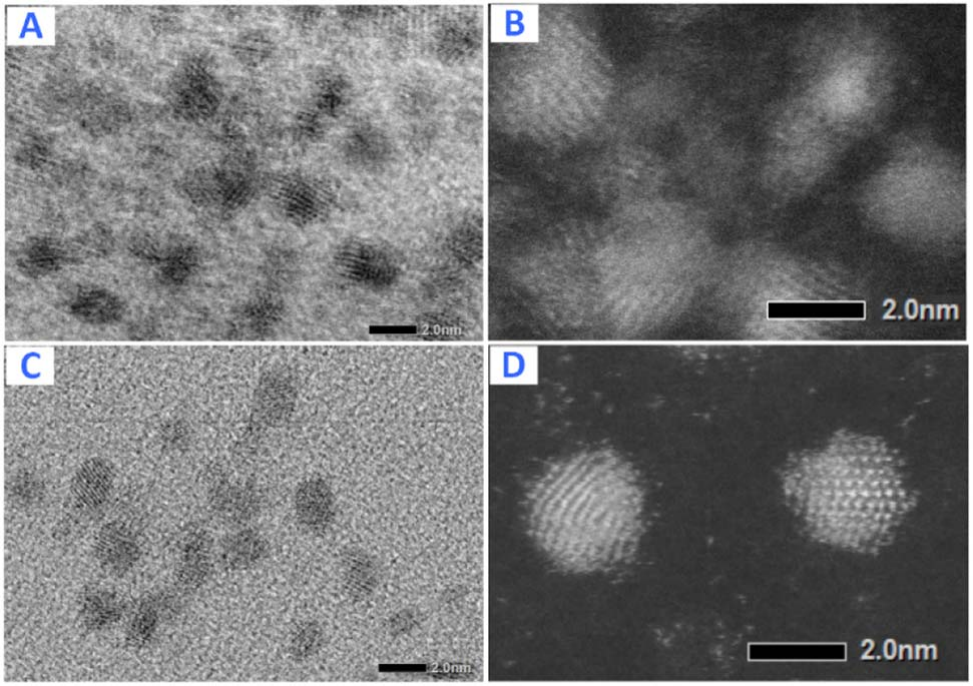
TEM images of Pt NPs with their corresponding bright-field images: before optimization (A and B) and after optimization (C and D).

It can be seen that the values of multiple response optimization are considerably lower than that of the individual result of single response optimization. These lower values happen because the yield and activity are competing with one another to determine the factors setting. The problem is fixed through the composite desirability of *D* = 0.821 to get the factors setting which optimizes both responses, *i.e.*, *A* = 3.5, *B* = 406.25, *C* = 0.5, and *D* = 0.45. The predicted maximum values of the responses are yield = 80.53% and activity = 8007 μmol s^−1^ g_Pt_^−1^ together with individual desirabilities of 1.000 and 0.674, respectively. The validation of yield and activity from multiple response optimization are shown in Table 4. The predicted yield and activity differences are 1.45% and 3.0%, respectively. The increased activity of the optimized Pt NPs can be contributed to less agglomeration as confirmed by TEM imaging (Fig. 16) of Pt NPs with their corresponding bright-field images: before optimization (*A* and *B*) and after optimization (*C* and *D*).

**Table 4:** Result of multiple response optimization for the synthesis yield and Pt-activity

Factor	Optimized				
	Factor	Yield (%)	Yield validated	Activity (μmol s^−1^ g_Pt_^−1^)	Activity validated
*A*: Pt in precursor (mg)	3.5	80.53	81.71	8007	8255
*B*: green reductant (mg)	406.25				
*C*: synthesis time (hr)	0.5				
*D*: surfactant fraction	0.45				

**Fig. 17:**
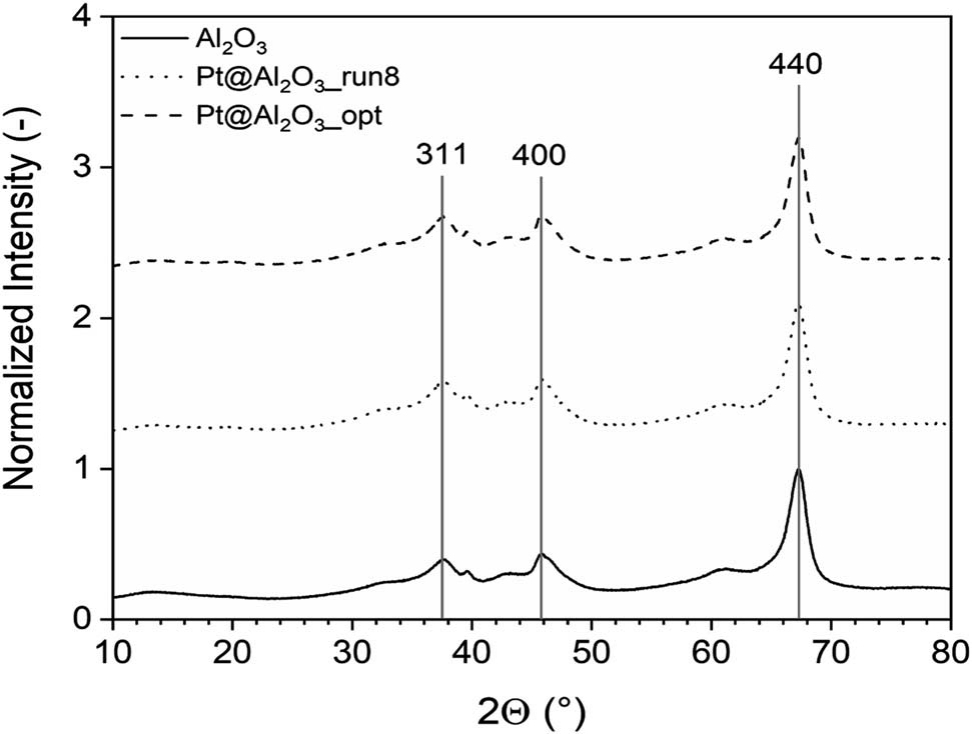
The XRD analysis of the unoptimized and optimized Pt/Al_2_O_3_ catalysts which shows three diffraction peaks for (311), (400), and (440) reflections.

The XRD study is depicted in Fig. 17 which shows three diffraction peaks for (311), (400), and (440) reflections of Al_2_O_3_ at about 37.6°, 45.7° and 66.6°, respectively (JCPDS card no. 10-0425). After deposition of platinum, the crystal structure of Al_2_O_3_ remains and additional similar peaks were obtained. This indicates that the diffraction of Al_2_O_3_ peaks overlay the Pt peaks which is also shown by several researchers,^34,35^ where the XRD peaks of Al_2_O_3_ and Pt/Al_2_O_3_ look the same.

The presence of Pt NPs on the Alumina is also proven by the BET analysis. The initial surface area of Al_2_O_3_ support of 155 m^2^ g^−1^ was slightly reduced after Pt deposition to 129 m^2^ g^−1^ and 132 m^2^ g^−1^ for Pt/Al_2_O_3__run8 (the most active catalyst before optimization) and Pt_Al_2_O_3__opt (the optimized catalyst), respectively. This reduction in surface area was expected, as Al_2_O_3_ is a porous material, and some platinum species are deposited into the porous matrix.

**Fig. 18:**
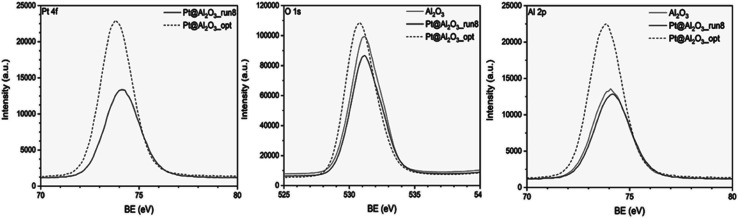
The XPS analysis of the unoptimized and optimized Pt@Al_2_O_3_ catalysts.

XPS was utilized to investigate the surface chemical environment of Al_2_O_3_ and Pt@Al_2_O_3_. The XPS pattern of Al_2_O_3_ shows the expected peaks and corresponding binding energies for Al 2p and O 1s. After immobilization of platinum nanoparticles onto the surface of Al_2_O_3_, the Pt 4f XPS pattern shows the same peaks and binding energies as for bare Al_2_O_3_ (Fig. 16). The problem is that Al 2p and Pt 4f have overlapping binding energies so that further evaluation of the electronic structure of Pt nanoparticles was not possible. This situation has already been reported in the literature.^34^ Therefore, only a small shift toward lower binding energies is observed for the Pt/Al_2_O_3__opt (Fig. 18).

## 4. Conclusion

The optimization of the green synthesis of supported Pt nanocatalyst *via* the microemulsion method has been demonstrated using design of experiment approaches supported by reactor testing and catalyst characterization. To arrive at the most efficient nanocatalyst synthesis, both the yield and catalytic activity were optimized simultaneously. A multiple response optimization was therefore used rather than a single response optimization, and the results of multiple response optimization were lower than those of the single responses because the yield and activity compete with one another to determine the factor settings. With the single optimization, the synthesis yield of supported Pt NPs can be increased from their average value of 78.9% to 99.75% and activity from 2136 to 15 600 μmol s^−1^ g_Pt_^−1^. The results of multiple response optimization to the yield and activity are 81.71% and 8255 μmol s^−1^ g_Pt_^−1^, respectively. Although the technique is applied to synthesizing supported Pt NPs, the principle of optimization presented in this study can also be used in other cases with different factors and response of interest.

## Conflicts of interest

The authors declare no conflicts of interest.

## Supplementary Material

RA-012-D2RA04134K-s001
