# Supplementary material for: Efficient preparation of nanocatalysts. Case study: green synthesis of supported Pt nanoparticles by using microemulsions and mangosteen peel extract
Source: RSC Adv. 2022 Nov 30;12(53):34346–58. doi: 10.1039/d2ra04134k (PMC9709592; doi:10.1039/d2ra04134k)
Supplement: RA-012-D2RA04134K-s001 [file RA-012-D2RA04134K-s001.pdf]

Electronic Supplementary Material (ESI) for RSC Advances.
This journal is © The Royal Society of Chemistry 2022

Supporting Information

Efficient Preparation of Nanocatalysts

Case Study: Green Synthesis of Supported Pt Nanoparticles by Using Microemulsions and Mangosteen Peel Extract

Riny Yolandha Parapat,[a,b] Michael Schwarze,[b] Alwin Ibrahim,[a] Minoo Tasbihi,[b] Reinhard Schomäcker[b]
[a]Chem. Eng. Dept. of Institut Teknologi Nasional Bandung, PHH. Mustopha 23, 40124 Bandung, Indonesia.
[b]Department of Chemistry, Technische Universität Berlin, Straße des 17. Juni 124, 10623 Berlin, Germany.

1. The complete TEM and EDX analysis of Pt NPs after synthesized by microemulsions

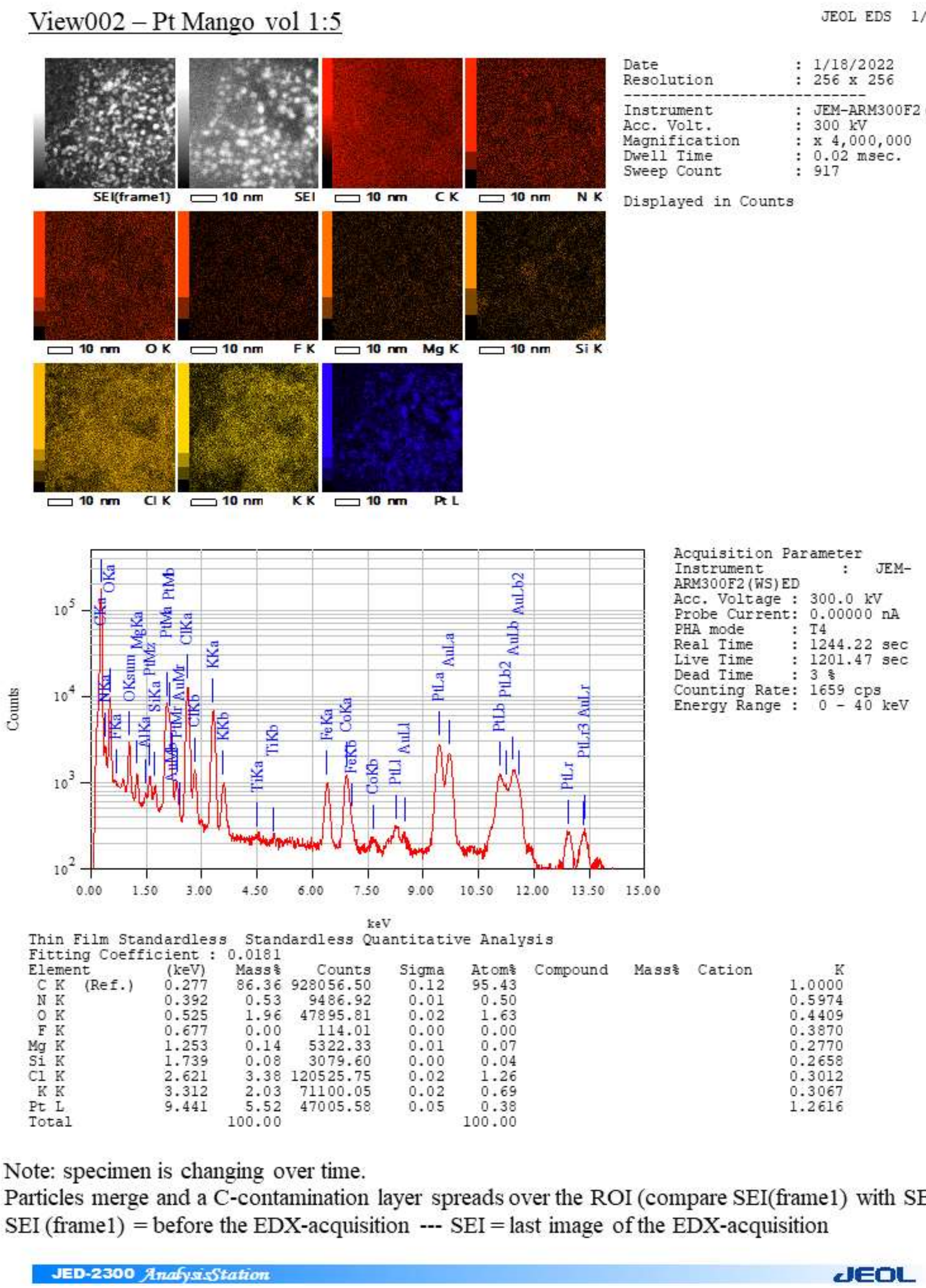

Fig. S1 Complete TEM - EDX investigation of Pt NPs in the microemulsion before deposited on Al_2O_3

2. Anova Tables for the Yield and Activity of Pt/Al_2O_3

The significant factors that affect the Yield are A, B and the CD interactions, where A, B, C and D are amount of Pt in precursor, amount of reductant, synthesis time, and mass fraction of surfactant, respectively.

Table 1. Analysis of Variance for Yield with α = 0.05

Source	DF	Adj SS	Adj MS	F-Value	P-Value
Model	14	2593.38	185.241	2.29	0.057
Linear	4	1750.45	437.612	5.41	0.006
**A**	1	989.15	989.146	12.22	**0.003**
**B**	1	454.67	454.673	5.62	**0.031**
C	1	218.60	218.599	2.70	0.120
D	1	88.03	88.029	1.09	0.312
Square	4	0.00	0.000	0.00	1.000
A*A	1	0.00	0.000	0.00	1.000
B*B	1	0.00	0.000	0.00	1.000
C*C	1	0.00	0.000	0.00	1.000
D*D	1	0.00	0.000	0.00	1.000
2-Way Interaction	6	842.93	140.489	1.74	0.177
A*B	1	155.88	155.875	1.93	0.184
A*C	1	246.96	246.961	3.05	0.100
A*D	1	5.19	5.187	0.06	0.803
B*C	1	23.16	23.160	0.29	0.600
B*D	1	48.70	48.703	0.60	0.449
**C*D**	1	363.05	363.045	4.49	**0.050**
Error	16	1294.60	80.913		
Total	30	3887.98			

The significant factors that affect the Pt Activity are A, B and the AD and BD interactions, where A, B, C and D are amount of Pt in precursor, amount of reductant, synthesis time, and mass fraction of surfactant, respectively.

Table 2. Analysis of Variance for Activity with α = 0.05

Source	DF	Adj SS	Adj MS	F-Value	P-Value
Model	14	14345759	1024697	4.52	0.003
Linear	4	9689018	2422255	10.68	0.000
**A**	1	7746048	7746048	34.16	**0.000**
**B**	1	1846802	1846802	8.14	**0.011**
C	1	93150	93150	0.41	0.531
D	1	3019	3019	0.01	0.910
Square	4	0	0	0.00	1.000
A*A	1	0	0	0.00	1.000
B*B	1	0	0	0.00	1.000
C*C	1	0	0	0.00	1.000
D*D	1	0	0	0.00	1.000
2-Way Interaction	6	4656741	776123	3.42	0.023
A*B	1	422500	422500	1.86	0.191
A*C	1	209764	209764	0.93	0.350
**A*D**	1	1365392	1365392	6.02	**0.026**
B*C	1	379764	379764	1.67	0.214
**B*D**	1	1795600	1795600	7.92	**0.012**
C*D	1	483720	483720	2.13	0.163
Error	16	3627961	226748		
Total	30	17973720			

2. FTIR analysis of Al_2O_3, Pt/Al_2O_3 and the Mangosteen Peel

The absence of any residual organic species on the Pt catalyst was confirmed by the FTIR analysis

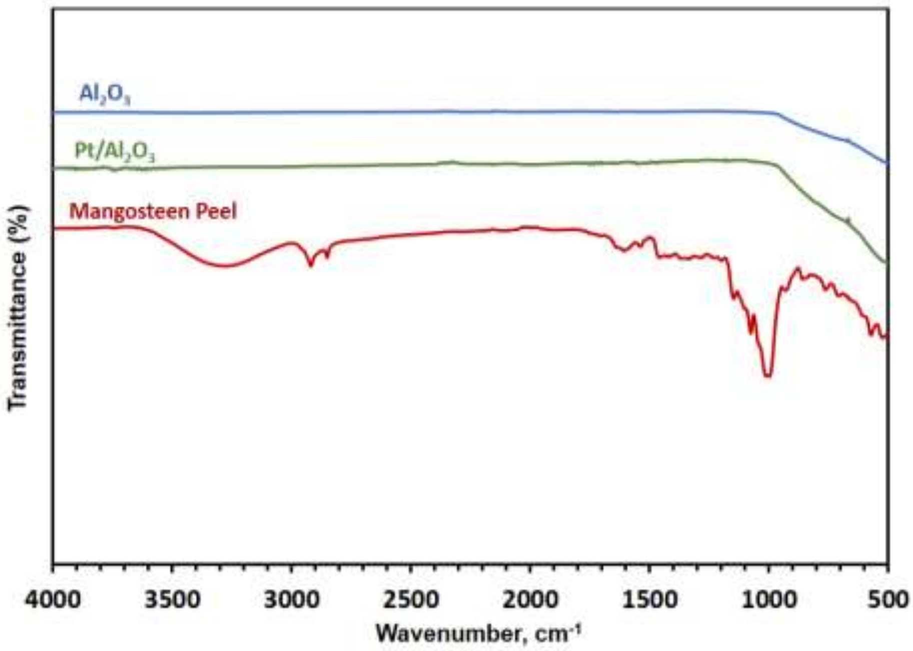

Fig. S2 FTIR analysis of Al_2O_3, Pt/Al_2O_3 and the Mangosteen Peel
